# Oral microbiota and autism spectrum disorder (ASD)

**DOI:** 10.1080/20002297.2019.1702806

**Published:** 2019-12-12

**Authors:** Ingar Olsen, Steven D. Hicks

**Affiliations:** a Department of Oral Biology, Faculty of Dentistry, University of Oslo, Oslo, Norway; b Department of Pediatrics, Penn State College of Medicine, Hershey, PA, USA

**Keywords:** Autism, oral microbiota, microbial oral-brain axis, dissemination of oral bacteria, neurological disorders

## Abstract

Autism spectrum disorder (ASD) is associated with several oropharyngeal abnormalities, including dysbiosis in the oral microbiota. Since the oral cavity is the start of the gastrointestinal tract, this strengthens and extends the notion of a microbial gut-brain axis in ASD and even raises the question whether a microbial oral-brain axis exists. It is clear that oral bacteria can find their way to the brain through a number of pathways following routine dental procedures. A connection between the oral microbiota and a number of other brain disorders has been reported. As the evidence so far for an association between the oral microbiota and ASDs rests on a few reports only, further studies in this field are necessary. The current review discusses a possible relationship between oral bacteria and the biologic and symptomologic aspects of ASD, focusing on the clinical implications for diagnostic and therapeutic development.

Autism spectrum disorder (ASD), which appears in the first years of life, is associated with abnormalities such as buccal sensory sensitivity, taste and texture aversions, speech apraxia and changes in salivary ribonucleic acid expression [1–9]. It has been estimated that one in 59 American children is affected by ASD, and there has been a marked increase in its incidence and prevalence over the last decades [10]. There are a number of co-occurring pathologies in ASD (Table 1). Early clinical interventions can improve symptom trajectory, but do not completely abrogate ASD symptoms, and pharmacologic interventions are limited. One novel avenue for diagnostic and therapeutic research is the emerging association between ASD and oral bacteria communities [1,11]. This review will discuss the possible relationship between oral bacteria and the biologic and symptomologic aspects of ASD, focusing particularly on the clinical implications for diagnostic and therapeutic development.

**Table 1.** Co-occurring diseases in ASD (from ref [29])

Brain-related comorbidities
Altered metabolite profile in urine and blood
Fragile X syndrome, Rett syndrome and tuberous sclerosis
Mitochondrial dysfunction
Gut-related co-morbidities
Gastrointestinal symptoms
Increased permeability of the intestinal epithelial barrier
Decreased expression of brush-border disaccharides in the intestinal epithelium
Other co-morbidities
Altered expression of tight junction protein in the BBL
Increased amounts of activated microglia cells

## Dental problems in ASD

Children with autism can have multiple medical and behavioral problems that make adequate oral hygiene and dental treatment difficult to perform. In a study of 61 children with ASD, aged 6–16 years (45 males and 16 females), higher caries prevalence, poor oral hygiene and extensive unmet needs for dental treatment compared to controls without autism were reported [12]. This could promote dissipation of oral bacteria to the circulation and potentially the brain [13], initiated by widespread dental plaque-induced diseases such as caries and gingivitis/periodontitis [14–16].

## Studies on oral bacteria in ASD

Qiao et al. [11] used high throughput sequencing to compare the oral microbiota in children with ASD to healthy controls (Table 2). Approximately 1 ml of non-stimulated, naturally outflowed saliva was first collected. Then, supragingival plaques were obtained separately from caries-free molars in four quadrants (upper right, upper left, lower right and lower left) per subject. The 111 samples were divided into four groups: 1) salivary samples from healthy controls (HS; n = 27); 2) dental samples from healthy controls (HP; n = 26); 3) salivary samples from ASD patients (AS; n = 32); 4) dental samples from ASD patients (AP; n = 26). The transcriptional activity of the salivary and dental microbiota in ASD patients differed markedly from that of healthy children. In children with ASD, a lower bacterial diversity was demonstrated than in controls, consistent with findings from the gut [17,18]. This finding was particularly pronounced in dental plaque samples. The genera *Haemophilus* in saliva and *Streptococcus* in dental plaque were significantly more abundant in ASD whereas *Prevotella, Selenomonas, Actinomyces, Porphyromonas* and *Fusobacterium* were reduced. A depletion of the *Prevotellaceae* family co-occurrence network was also detected in plaque from ASD patients. In saliva, no phylotypes were highly correlated with decayed, missing, filled teeth or surfaces (DMFT/S). In dental plaque, however, six phylotypes including *Streptococcus, Actinomyces* and *Capnocytophaga* were positively associated with DMFT/S. Accordingly, presence of dental caries was more related to the microbiota of dental plaque than to that of saliva. *Aggregatibacter segnis* (OTU220) was positively associated with bleeding on probing, gingival index and periodontitis. The bacterial patterns observed in individuals with ASD suggested a possible role for microorganisms in this disorder, but did not establish a causal relationship. The results also suggested that aversion of ASD patients to dental hygiene interventions might be one mechanism for oral dysbiosis.

**Table 2.** Clinical trials performed on the oral microbiota in children with autism spectrum disorder (ASD)

Authors/ref	Age (yrs)	Method	Groups	Results and Conclusions
Hicks et al. [1]	2–6	RNA extraction & Shotgun sequencing Genetic activity of oral microbiota were examined	ASD (n = 180) DD (n = 60) TD (n = 106)	12 taxa were altered between groups 28 taxa distinguished ASD patients with and without GI disturbances 5 microbial ratios distinguished ASD from TD 3 microbial ratios distinguished ASD from DD GI microbe disruption in ASD extended to pharynx Oral microbiome profiling has a potential to evaluate ASD status
Qiao et al. [11]	7–14	High throughput sequencing	ASD (n = 32) Controls (n = 27)	Salivary and dental microbiota distinct from those of controls Lower bacterial diversity in ASD than in controls, especially in dental samples *Haemophilus* (saliva) and *Streptococcus* (plaques) were significantly higher in ASD, while commensals like *Prevotella, Selenomonas, Actinomyces, Porphyromonas* and *Fusobacterium* were reduced Depletion of *Prevotellaceae* co-occurrence network detected in ASD (dental plaque) Distinguishable bacteria were correlated with clinical indices (disease severity and oral health status; dental caries) Diagnostic models based on key microbes constructed with 96.3% accuracy in saliva The habitat-specific profile of the oral microbiota in ASD may help diagnosis of ASD

ASD = autism spectrum disorder; DD = developmental delay; TD = typically developing

In a second study [1], changes in the salivary microbiome of children 2–6 years old were identified across three developmental profiles: ASD (n = 180), non-ASD with development delay (DD; n = 60) and typically developing (TD; n = 106) children (Table 2). Actively transcribing taxa were quantified and tested for differences between the groups and within ASD endophenotypes. Between the developmental groups, 12 bacterial taxa differed. Of particular note, 28 taxa were distinctly active among ASD patients with gastrointestinal (GI) disturbance. By group classification, five microbial ratios distinguished ASD from TD children (79.5% accuracy), three separated ASD from DD (76.5% accuracy) and three identified ASD children with GI disturbance from ASD peers without GI comorbidities (85.7% accuracy). There were significant differences in microbial transcription of energy metabolism and lysine degradation pathways across the ASD, TD and DD groups. The results indicated that GI microbial disruption in ASD likely extends to the oropharynx. Given the largely unidirectional transit of bacteria from the oropharynx to the lower GI tract, this implies that oral dysbiosis may actually serve as a primary source for a portion of the fecal dysbiosis reported in numerous ASD studies [19–21].

## Oral microbiota affecting the intestine

Studies in animals and humans have demonstrated that oral bacteria can be transferred to the gut, changing its microbial composition and perhaps even host immune responses [22–24]. Oral bacteria and stool bacteria overlapped in almost half (45%) of the subjects in the Human Microbiome Project [25]. The ectopic transfer of oral bacteria has also been reported in patients with systemic diseases, such as inflammatory bowel disease [26]. Co-occurring GI problems are common in children with ASD [27]. GI symptoms were four times more prevalent in children with ASD than in children with typical development [28]. The GI symptoms seen in individuals with autism can include constipation, diarrhea, bloating, abdominal pain, reflux, vomiting, gaseousness and foul-smelling stools (for a review see [29]). Such symptoms may be related to the lower bacterial diversity reported in children with ASD [17].

Ectopic transfer of oral bacteria can occur in patients with localized ‘chronic’ periodontitis. *Porphyromonas gingivalis*, which is proposed as a keystone bacterium in this disease [30,31], causes dysbiosis in the periodontal microbiota. This may lead to microbial dysregulation in the gut since each day 10^8–^10^10^ of *P. gingivalis* can be swallowed [32,33]. Changes in the gut microbiota composition could induce permeability of the gut barrier and immune activation leading to systemic inflammation. Ectopic colonization of oral bacteria in the intestines has been found to drive T-helper (TH)-1 cell induction and inflammation [22]. In one case study, *Klebsiella* spp. isolated from the saliva of a patient with inflammatory bowel disease were marked inducers of TH-1 cells. Ongoing colonization by oral bacteria was suggested to perpetuate gut microbiota dysbiosis and chronic inflammation. In this setting, the oral cavity can serve as a reservoir for potential intestinal pathobionts that aggravate intestinal disease. Wang et al. [27] and Ashwood [34] have also reported abnormalities in intestinal immunity in children with ASD.

## Dysbiosis of the intestinal microbiota

Dysbiosis of the intestinal microbiota is an emerging etiological factor proposed for ASD [17,29,35–40]. The GI microbiome is thought to influence host behavior and neurodevelopment through the ‘microbial-gut-brain axis’ [41,42]. Imbalance in the intestinal microbiota or its metabolites may affect several complex behaviors (such as emotional and anxiety-like behaviors), and influence brain development or modulate cognition [11,43–45]. A microbiota-gut-brain axis is based on a bidirectional physiologic connection where information between the host microbiome, gut and brain are exchanged [46]. This likely involves cross talk between the central nervous system and microbes within the GI tract through direct neural activation, immune modulation, and hormonal, peptidergic and epigenetic signaling [47–50]. Below, we consider how each of these factors may be translated to an ‘oral-brain axis’.

## Oral microbiota and the brain

### How oral microbiota may reach the brain

**Figure 1.**
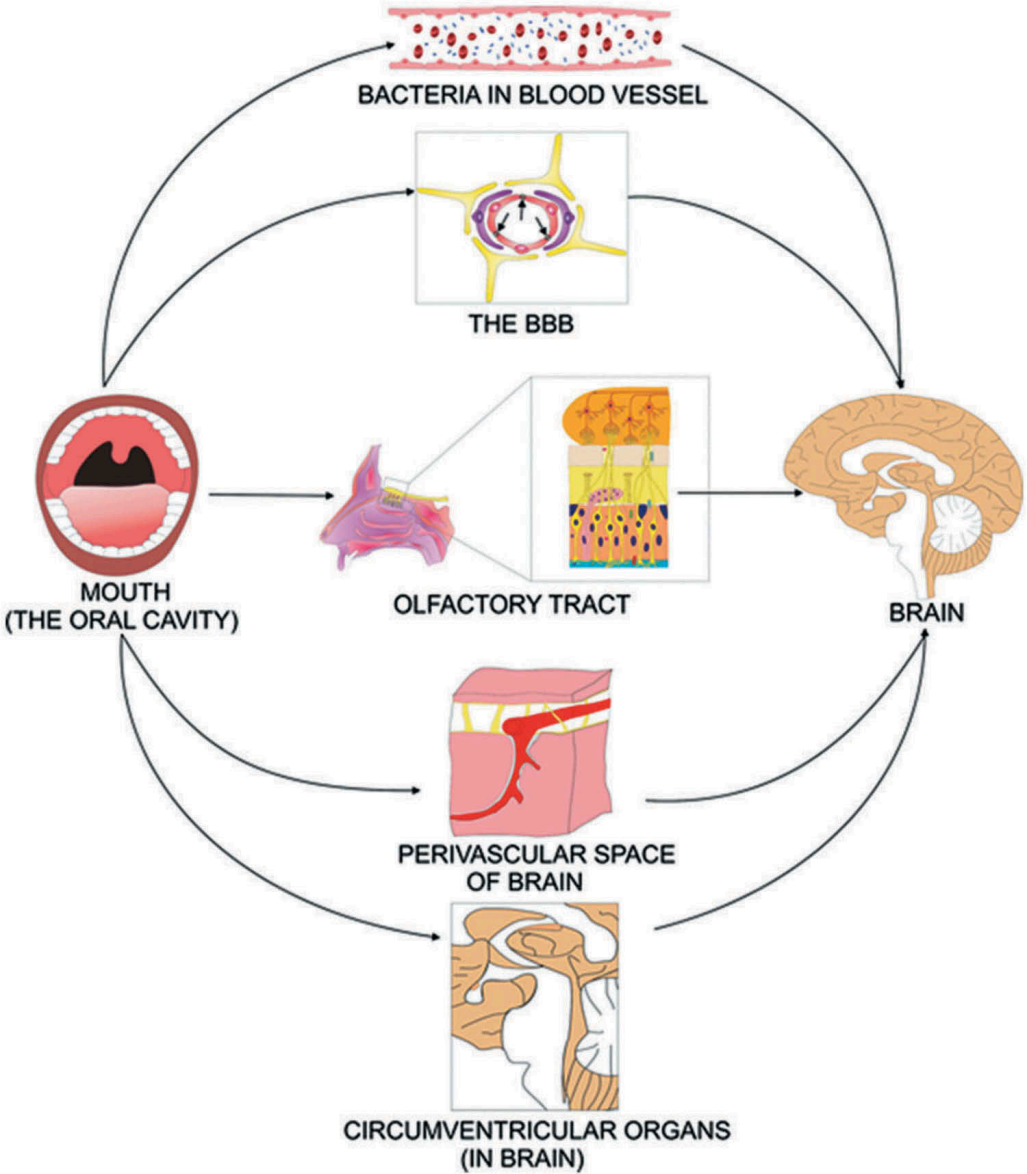
Direct and indirect mechanisms of infecting the brain. In the direct mechanism, the oral cavity infects the olfactory tract, and the olfactory nerve transfer the bacteria to the brain. In other mechanisms, bacteria inside the mouth infect the blood and find their way via blood, blood–brain barrier (BBB), perivascular spaces and circumventricular organs to the brain (figure is based on concepts presented in ref [51] and collected from ref [55])

There are several plausible pathways for bacteria in the mouth to reach the brain and directly influence neuro-immune activity and inflammation [51] (Figure 1). Even routine dental procedures can cause bacteremia [52], and a portion of these microbes may traverse the blood–brain barrier (BBB). Altered transcript expression has been described in microglia of ASD individuals, and disrupted microglia function could impair BBB integrity [53]. Increased permeability of the BBB has been described in children with ASD [54]. This could expose the brain to bacterial metabolites, thereby triggering an inflammatory response and altering metabolic activity within the central nervous system [29]. Prolonged disruption of energy metabolism within neurons, oligodendrocytes and glia could lead to structural changes in the cortex, hippocampus, amygdala or cerebellum, which have all been documented in ASD individuals [29].

### How oral microbiota may affect the brain: inflammation

**Figure 2.**
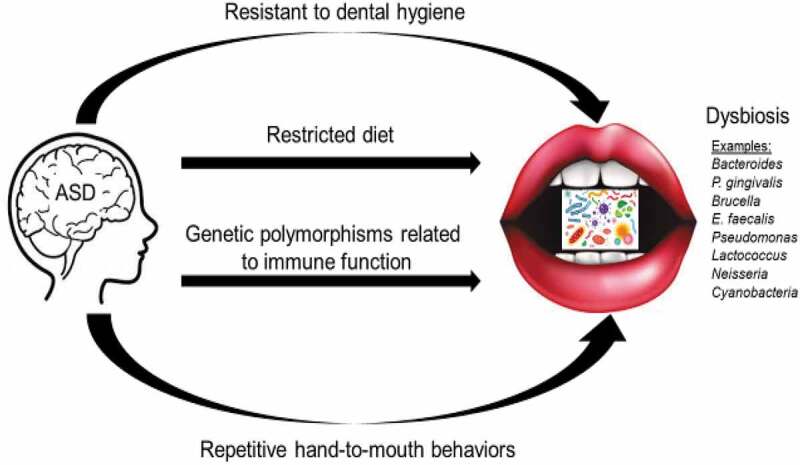
ASD phenotype can lead to oral dysbiosis

Central nervous system inflammation has been a prominent feature in studies of both animal models and post-mortem brains from individuals with ASD. For example, a study by Morgan and colleagues described the up-regulation of microglia in the ASD brain [56]. Cytokines and chemokines are also elevated in the cerebrospinal fluid of ASD patients [57,58]. Moreover, genes associated with immune and inflammatory responses are activated in the ASD cortex [59]. There appears to be a general dysregulation of the immune system towards a pro-inflammatory phenotype in ASD individuals [58,60]. Such inflammation in the developing brain may lead to synapse malfunction [61]. A significant reduction of both synaptic transmission and excitability has been observed when hypoxia and inflammation occur in combination, whereas re-oxygenation leads to neuronal hyper-excitability [62]. Malfunctioning synapses may cause the release of vasopressin, which has been shown to affect social behavior [61]. Interestingly, induction of inflammation early in gestation may promote an ASD-like phenotype through increased synaptic excitation [58] (Figure 2). In this process, early life exposure to inflammation might prime microglial cells to become hyper-responsive to subsequent insults [63]. Notably, chronic application of periodontal pathogens in mice have resulted in the development of neuropathological changes consistent with Alzheimer’s disease (a condition in which cortical inflammation is a decisive factor) [64]. Oral bacteria reaching the brain could reduce the anti-oxidative capacity and lead to reduction in the ability of mitochondria to produce energy in ASD individuals [65]. Gram-negative, putative periodontal pathogens, are rich in lipopolysaccharide (LPS) which has pro-inflammatory activity. Leakage of LPS through the BBB in ASD individuals could lead to inflammation in the central nervous system. Furthermore, increased levels of LPS in individuals with autism have been found to correlate with high levels of IL-6, a pro-inflammatory cytokine [66].

### How oral microbiota may affect the brain: metabolic alterations

**Figure 3.**
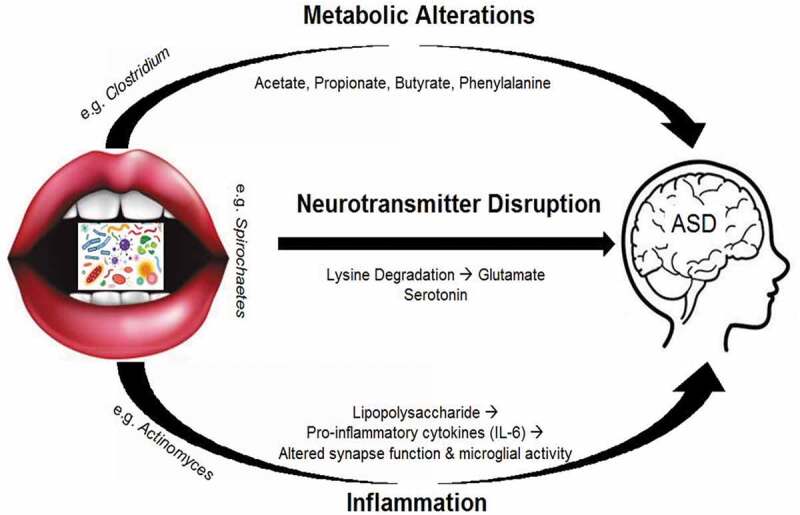
Proposed mechanisms for development of ASD

Microbial communities have a significant impact on metabolism within the human GI tract [67,68]. Thus, oral dysbiosis in ASD could lead to disruptions in the metabolome – a putative mechanism for ASD pathogenesis [69–71]. There are indications that increases in acetate and propionate, as well as decreases in butyrate (short-chain fatty acids of bacterial origin), can be involved in the development of ASD together with indoles [29] (Figure 3). There are also increased levels of 3-(3-hydroxyphenyl)-3-hydroxypropionic acid, 3-hydroxyphenylacetic acid and 3-hydroxyhippuric acid in children with ASD, which together indicate potential perturbations in the phenylalanine metabolism [72]. These metabolites are related to the abundance of *Clostridium* spp. and associated with aggravated restricted and repetitive behaviors in children. The high abundance of intestinal *Clostridium* detected in ASD may reflect a pathogenic role for these particular organisms [73]. Whether metabolic changes result from the *oral* microbiota composition in children with ASD remains to be determined. However, a study of oral microbe transcription across 346 children (including 180 with ASD) identified ASD-specific changes in pathways involving lysine degradation – a precursor to the neurotransmitter glutamate, that has been implicated in ASD pathogenesis [1]. By using saliva samples from this same cohort, the authors also described ASD-specific alterations in human microRNA expression that were associated with microbial activity, and implicated in cell growth and metabolism pathways [1]. Such findings provide a framework for human–microbial interaction at the biochemical level that may have functional consequences for host behavior.

## Cause–effect relationship between ASD and microbes?

Although numerous studies have identified microbial disruptions in patients with ASD and linked those disruptions to symptoms and behavior, we still do not fully understand the mechanism by which microbial communities are dysregulated in individuals with ASD. Furthermore, it is unclear if the microbial patterns described in individuals with ASD cause ASD symptoms, or result from behaviors common to the ASD phenotype.

### Phenotype

Microbial dysbiosis may be influenced by the ASD phenotype. This could occur through resistance to dental hygiene, lack of a varied diet, and placing objects into the mouth as sensory seeking behavior. Discontinuation of oral hygiene in 29 orally healthy individuals for 4, 7 and 10 days, and assessment 14 days after resumption of oral hygiene, was associated with a significant increase in relative abundance of potential cariogenic *Leptotrichia* species and a decrease in *Streptococcus* species [74]. This study demonstrated the importance of regular oral hygiene on the maintenance of oral homeostasis. Furthermore, dental caries can be caused by ecological imbalance of commensal microbiota (mainly due to lack of a varied diet, such as frequent carbohydrate consumption) [75]. Placing foreign objects (e.g. toys, dirt, etc.) in the mouth is yet another source of dysbiosis, because these objects can be contaminated with microorganisms from unwashed hands in contact with other human body fluids [76].

Discerning the importance of ASD phenotype as a modulator of the oral and intestinal microbiota will likely require parallel studies in both humans and animal models. One potential strategy to help elucidate the cause/effect dilemma involving microbial disruption and ASD, is to establish ASD in a gnotobiotic animal and examine the potential of major members of the oral and intestinal microbiota (for example, oral *P. gingivalis* and *Klebsiella* spp. [22,77], and intestinal *Clostridium* spp. [17]) to induce ASD symptoms. Furthermore, promising pilot studies on microbiota transfer therapy (i.e. fecal transfer) should be extended to include double-blinded placebo controlled trials with well-defined a-priori hypotheses for functional outcome measures. Studies carefully examining the interaction between probiotic therapies (e.g. *Bifidobacterium*) and antibiotic therapies (e.g. vancomycin, minocycline) may also provide useful information about whether microbial modulation can alter ASD behaviors [78,79].

### Environmental and genetic factors

Although most cases of ASD are idiopathic [17], both environmental and genetic factors are likely important for ASD development [80,81]. Exposure to environmental risk factors or genetic risk transmission can affect the maternal microbiome [21]. Offspring acquires a large portion of their microbiome from mothers during the birth process. Whether birth occurs via the vaginal canal or by cesarean section significantly affects the infant’s microbiome [82–84]. Thus, delivery mode might play a role in certain neurodevelopmental disorders. To date, studies examining the relationship between ASD and cesarean sections have demonstrated mixed results [85–87]. Changes in the microbiome due to stress might also be transferred to offspring during birth, initiating microbial dysbiosis that lasts into adulthood [88–90]. It has been reported that early life exposures to plastics and other chemicals can affect the infant microbiota [21]. Disentangling the relationship between these exposures, microbiome profiles and developmental trajectories is a difficult task that will require careful, comprehensive data collection, and powerful statistical models that can account for the interplay of many different environmental factors.

## Clinical implications for microbial dysbiosis in ASD

### Biomarkers

Many children with ASD exhibit hyperserotonemia [91], augmented oxidative stress [92] and increased expression of neuro-inflammatory markers [93–95]. Such disturbances may be related to disruptions in glutamate [96] and brain-derived neurotrophic factor [97]. In a study using mass spectrometry, West et al. [98] identified several blood plasma metabolites that could be of value in diagnosing ASD in 4 to 6 years old children. Amino acid metabotypes have been proposed as biomarkers for diagnostic subtypes of ASD [99]. Metabolites detected in blood and urine such as short-chain fatty acids, indoles and LPSs of bacterial origin might have diagnostic utility [29]. However, this biologic approach has not demonstrated an ability to differentiate children with ASD from peers with non-ASD developmental delay – a comparison that forms the crux of the ASD diagnostic dilemma. At the present time, the diagnosis of ASD remains dependent on clinical evaluation of behavioral symptoms, with no laboratory or objective biologic tests [100]. There is, however, growing evidence that oral microbes may be useful as a diagnostic aid in ASD [1,11]. This is an extension of a larger body of evidence relating ASD to the gut microbiome. Recently, salivary poly-omic RNA measurement was described as a novel approach to accurately identify children with ASD [101]. This objective, quantitative algorithm accurately discriminated children with ASD from peers with either developmental delay, or typical development. It could one day be used as a rapid, biologic aid for ASD diagnosis. This would constitute an important advancement, given the evidence that early diagnosis and intervention lead to the improvement of developmental trajectories for children with ASD.

### Therapeutics

In animal studies, the microbiome has been shown to modulate social behavior through dysbiosis, while microbiome restoration may ameliorate ASD symptoms [102,103]. Hsaio et al. [36] demonstrated that microbial shifts within the gut of a maternal immune activation (MIA) mouse model that is known to display features of ASD, changed metabolites in the serum and that these caused autism-like behaviors. Notably, administration of a beneficial bacterium, *Bacteroides fragilis*, reversed the observed physiological, neurological and immunological anomalies. Wang et al. [104] reported that oral probiotics prevented ASD-like behaviors in offspring induced by maternal immune activation. *Bifidobacterium* (e.g. *B. longum, B. breve* and *B. infantis*) and *Lactobacillus* (e.g. *L. helveticus* and *L. rhamnosus*) are commonly employed probiotics in human patients. These probiotics have demonstrated promising effects on behaviors such as anxiety, depression, ASD, obsessive-compulsive disorder, and memory (including spatial and non-spatial memory) [105]. In a recent review by Ng et al. [106] it was concluded that prebiotics played a limited role in alleviating the GI and behavioral symptoms in children with ASD, but when combined with an exclusion diet (gluten and casein-free) could potentially impact sociability. Significant support for a microbial-gut-brain axis in ASD arises from studies demonstrating that microbiota transfer therapy changes the gut ecosystem and improves gastrointestinal and autism symptoms in children [107]. When microbiota transfer therapy was combined with antibiotics, bowel cleanse, and a stomach-acid suppressant, 18 individuals with ASD demonstrated significant improvements in GI symptoms, autism-related symptoms, and gut microbiota [108]. Follow-up 2 years after treatment found that most improvements in GI symptoms were maintained, and some ASD-related symptoms also remained improved. Notably, there was a significant increase in bacterial diversity and relative abundances of bifidobacteria and *Prevotella*. Well-designed, randomized, placebo-controlled clinical trials are needed to assess the effectiveness of probiotics and microbial transfer therapies in the treatment of ASD. Choice of appropriate strains, dose, and timing of treatment are all important factors to consider [109].

## Conclusions

Microbial studies of ASD have focused largely on fecal samples [45]. It is worth noting that the oral microbiota and a possible microbial oral-brain axis have been disregarded in this context. The mouth is an extension of the digestive tract and has an abundant microbiome that includes more than 700 identified bacterial species (http://www.homd.org). Oral bacteria can enter the circulation and cause bacteremia following routine procedures such as chewing, flossing, brushing and dental cleaning [52]. Oral microbiota may contribute to several neurological diseases, including Alzheimer’s disease [51,77,110–113], epileptic seizures [114], multiple sclerosis [115], migraines [116], and Parkinson’s disease [117–119]. Whether a microbial oral-brain axis exists in ASD has yet to be definitively demonstrated. However, the relationship of oral bacteria with neurological function makes the existence of such an axis highly plausible.
